# Synthesis of Novel Aryl(heteroaryl)sulfonyl Ureas of Possible Biological Interest

**DOI:** 10.3390/molecules15031113

**Published:** 2010-02-26

**Authors:** Franciszek Sączewski, Anna Kuchnio, Monika Samsel, Marta Łobocka, Agnieszka Kiedrowska, Karolina Lisewska, Jarosław Sączewski, Maria Gdaniec, Patrick J. Bednarski

**Affiliations:** 1 Department of Chemical Technology of Drugs, Faculty of Pharmacy, Medical University of Gdańsk, 80-416 Gdańsk, Poland; 2 Faculty of Chemistry, A. Mickiewicz University, 60-780 Poznań, Poland; 3 Department of Pharmaceutical and Medicinal Chemistry, Institute of Pharmacy, University of Greifswald, D-17487 Greifswald, Germany

**Keywords:** 4-dimethylaminopyridinium arylsulfonylcarbamoylides, 4-dimethylamino-pyridinium arylsulfonyl carbamates, arylsulfonyl ureas, heteroarylsulfonyl ureas, arylsulfonyl isocyanate substitutes

## Abstract

The course of reaction of aryl and heteroaryl sulfonamides with diphenylcarbonate (DPC) and 4-dimethylaminopyridine (DMAP) was found to depend on the p*K*a of the sulfonamide used. Aryl sulfonamides with p*K*_a_ ~ 10 gave 4-dimethylamino-pyridinium arylsulfonyl-carbamoylides, while the more acidic heteroaryl sulfonamides (p*K*_a_ ~ 8) furnished 4-dimethylaminopyridinium heteroarylsulfonyl carbamates. Both the carbamoylides and carbamate salts reacted with aliphatic and aromatic amines with the formation of appropriate aryl(heteroaryl)sulfonyl ureas, and therefore, can be regarded as safe and stable substitutes of the hazardous and difficult to handle aryl(heteroaryl)sulfonyl isocyanates.

## 1. Introduction

Arylsulfonyl ureas constitute a well known class of compounds which exhibit a wide range of biological activities. The most important include: antidiabetic drugs (e.g., *glibenclamide*) [1], diuretic drugs (e.g., *torasemide*) [2], inhibitors of thromboxane synthase and thromboxane A2 receptor antagonists with antithrombotic properties [3,4] and inhibitors of acetohydroxyacid synthase (AHAS) which are used as herbicides (e.g., chlosulfuron) or agents active against *Mycobacterium tuberculosis* [5,6] as well as antiischemic [7], antimalarial [8], antifungal [9] and oncolytic (e.g., *sulofenur*) [10] agents. Of special interest are antagonists of chemokine receptors (CXCR2 receptors) which are potential drugs for the treatment of acute respiratory distress syndrome, asthma, chronic bronchitis, pulmonary fibrosis and cystic fibrosis [11]. Therefore, for medicinal chemistry purposes an easy access to arylsulfonylureas is of great importance. 

**Scheme 1:**
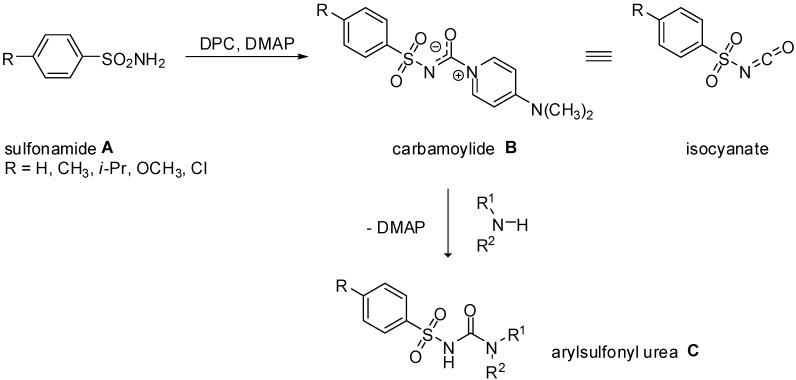
4-Dimethylaminopyridinium arylsulfonyl carbamoylides as stable substitutes of arylsulfonyl isocyanates.

Recently, we have described a facile method for the preparation of arylsylfonyl ureas of general formula **C** (Scheme 1) using 4-dimethylaminopyridinium arylsulfonyl-carbamoylides **B**, which constitute non-hazardous substitutes of arylsulfonyl isocyanates [12,13]. As shown in Scheme 1, the method consists in the reaction of aromatic sulfonamides **A** with diphenyl carbonate (DPC) in the presence of 4-dimethylaminopyridine (DMAP), followed by the reaction with primary or secondary aliphatic or aromatic amine. The above procedure worked well with phenylsulfonamide and its *para*-substituted congeners, such as *p*-alkyl, *p*-methoxy- and *p*-chloro-phenylsulfonamide as the substrates. The unique structure of the carbamoylides obtained were confirmed by IR and NMR spectra as well as single crystal X-ray structure analysis [13]. Carbamoylides **B** compose of an appropriate arylsulfonyl isocyanate and a DMAP molecule. The stability of these highly polarizable adducts is mainly due to the delocalization of the positive charge on the pyridine ring and the negative charge on the arylsulfonylcarbamoyl moiety.

In order to explore the scope of this procedure, an analogous previously not attempted reaction sequence starting from variously substituted phenylsulfonamides, naphthylsulfonamide as well as heteroarylsulfonamides, such as 2-thienyl- and benzothiazol-2-yl-sulfonamide has now been attempted.

## 2. Results and Discussion

**Scheme 2:**
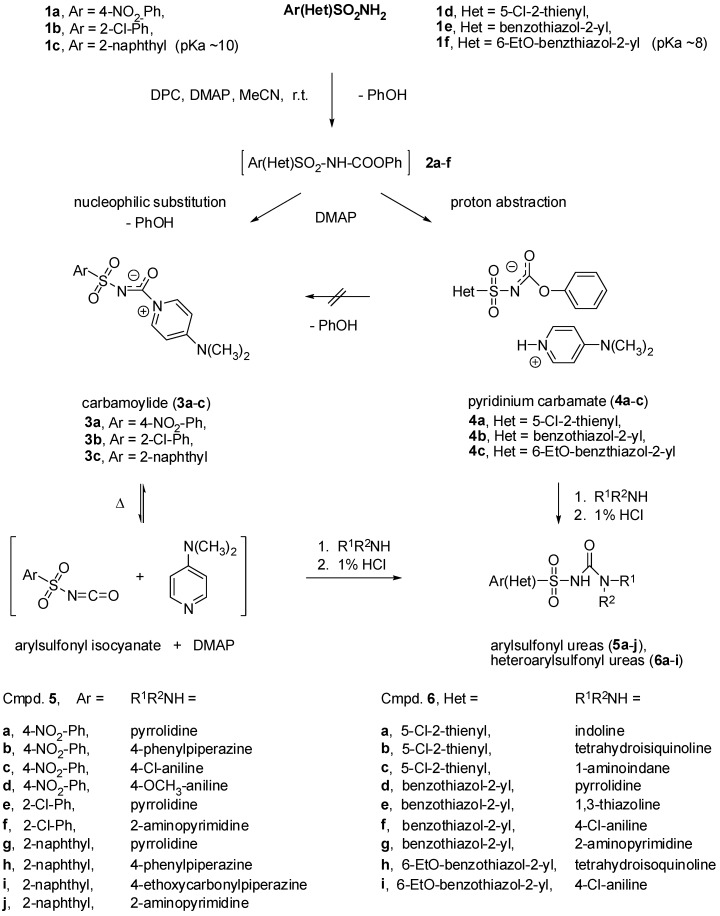
Synthesis of compounds **3**-**6**.

The results of the investigations are presented in Scheme 2. First, it was found that the reaction of variously substituted phenylsulfonamides **1a**-**c** (including *p*-nitrophenyl-, *o*-chlorophenyl- and 2-naphthylsulfonamide) with DPC in the presence of DMAP proceeded smoothly at room temperature to give appropriate carbamoylides **3a**-**c** in good yields. The reactions of these stable arylsulfonyl isocyanates substitutes carried out in CH_3_CN with both aliphatic and aromatic amines at elevated temperature afforded the desired arylsulfonyl ureas, which could be easily separated from the reaction mixtures upon treatment with 1% aqueous HCl.

However, when 2-thienyl and benzothiazol-2-yl-sulphonamides **1d**-**f** were treated with DPC in the presence of DMAP, the pyridinium carbamates **4a**-**c** were obtained as the sole products. Neither prolonged reaction times nor elevated temperatures changed the reaction course. 

**Figure 1:**
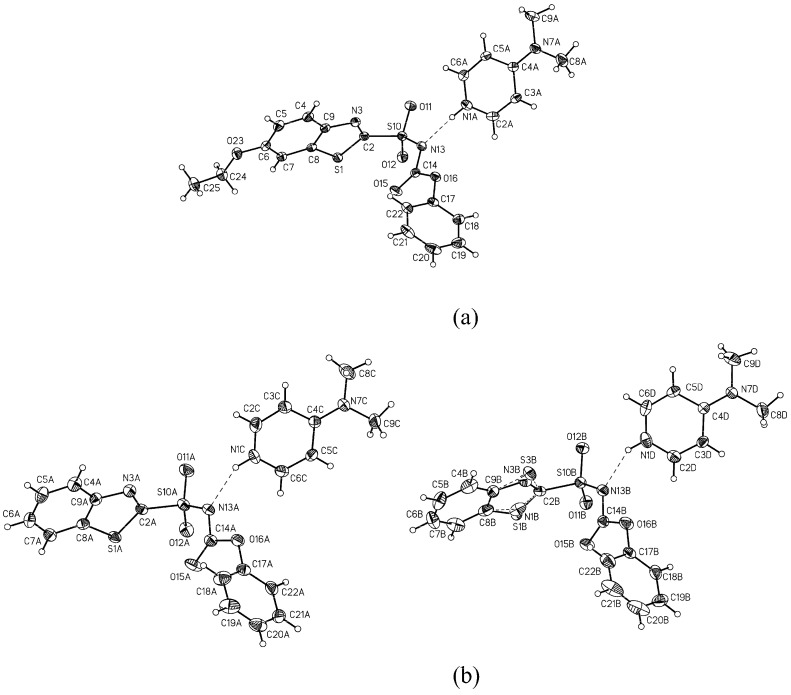
ORTEP drawings of (a) **4c** and (b) two symmetry independent molecules of **4b **with the atom labeling scheme. Displacement ellipsoids are drawn at the 50% probability level.

The structures of pyridinium carbamoylides **3** and pyridinium carbamates **4** were confirmed by IR and NMR spectroscopic data as well as X-ray single crystal structure analysis of **4b** and **4c** (Figure 1). The compounds **4b **and **4c **are organic salts with the proton transferred from the sulfonylcarbamate group to 4-dimethylaminopyridine. In the crystal the anions and the pyridinium cations form ionic pairs via N-H^+^···N^-^ hydrogen bonds. The anions assume similar conformation with one of the SO_2_ group O atoms approximately in the plane of benzothiazole moiety and the bond lengths and angles are as expected. The crystal packing is, to a large extent, governed by electrostatic interactions, with a pair of 4-dimethylaminopyridinium cations stacked in anti-parallel manner and completely surrounded by the anions.

The major difference between the two classes of sulfonamide derivatives studied lies in their relative acidity, hence, it was reasoned that the more acidic heteroarylsulfonyl sulfonamides **1d**-**f** (p*K*_a_ ~ 8) [14] formed the more acidic carbamates **2d**-**f** which, in turn, suffered proton abstraction by DMAP to give pyridinium salts of carbamates **4a**-**c**, while the less acidic arylsulfonamides **1a**-**c** (p*K*_a_ ~ 10) [15] gave rise to the formation of less acidic carbamates **2a**-**c** which underwent nucleophilic substitution reaction with DMAP to give the desired carbamoylides **3a**-**c**.

**Scheme 3:**
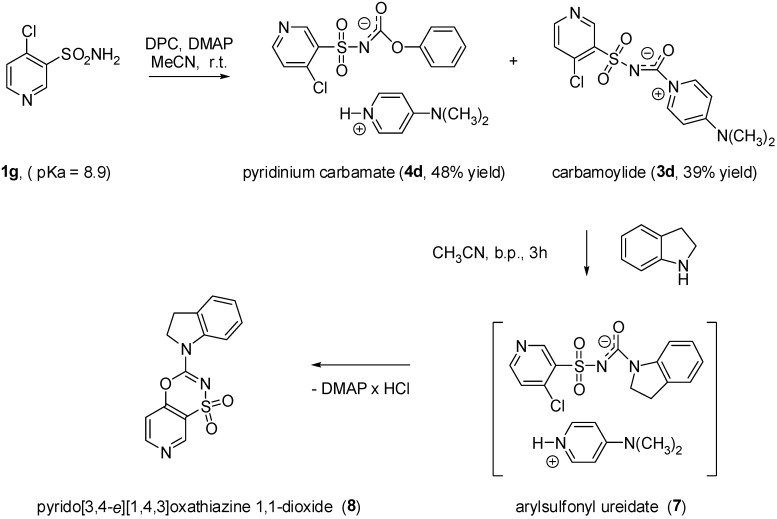
Synthesis of compound **8**.

In order to confirm the above hypothesis the reaction of DPC/DMAP couple with 4-chloropyridin-3-yl-sulfonamide (**1g**), characterized by a p*K*_a_ value of 8.9 [16], was performed. As shown in Scheme 3, the above reaction carried out at ambient temperature afforded two products: the carbamate pyridinium salt **4d** and the carbamoylide **3d** which could be separated from the reaction mixture by fractional crystallization in 48% and 39% yield, respectively. Interestingly enough, while the desired aryl(heteroaryl)sulfonyl ureas **5** and **6** were obtained in good yields from the reactions of either **3a**-**c** or **4a**-**c** with aliphatic, aromatic and heteroaromatic amines, upon treatment of both the carbamate **4d** and carbamoylide **3d** with an excess of a secondary amine, 3-(indolin-1-yl)pyrido[3,4-*e*] [1,4,3]oxathiazine 1,1-dioxide (**8**) was obtained. This might be formed as a result of an intramolecular nucleophilic substitution reaction in the transiently formed arylsulfonyl ureidate **7** (Scheme 3).

All the newly prepared compounds **5**, **6** and **8**, including benzothiazol-2-yl analogues of *sulofenur*, were screened *in vitro* for their potential cytotoxic activity using human urinary bladder cancer 5637, small cell lung cancer A-427 and large cell lung cancer LCLC-103H cell lines. None of these compounds exhibited cytotoxic activity at concentrations below 20 μM.

## 3. Experimental

### 3.1. General

Melting points were measured on a Boetius 545 apparatus and are not corrected. The IR spectra were obtained on a Nicolet 380 FTIR spectrometer using potassium bromide pellets and the frequencies were quoted in cm^-1^. The ^1^H-NMR spectra were recorded on a Varian Gemini spectrometer at 200 MHz or a Varian Unity Plus apparatus at 500 MHz. The chemical shifts (δ) are expressed in ppm in relation to tetramethylsilane as a standard and the coupling constants (*J*) are given in Hz. The starting substrates were commercial reagents.

### 3.2. General procedure for the preparation of 4-dimethylaminopyridinium arylsulfonyl carbamoylides ***3a-c*** and 4-dimethylaminopyridinium heteroarylsulfonyl carbamates ***4a-c***

A solution of appropriate arylsulfonamide **1a**-**c** (33 mmol) in acetonitrile (40 mL) was treated with 4-dimethylaminopyridine (DMAP, 4.1 g, 66 mmol) and the reaction mixture was stirred at room temperature until sufonamide **1** had dissolved. Then diphenyl carbonate (DPC, 2.3 g, 37 mmol) was added and the reaction mixture was left overnight at room temperature. The solid that precipitated was separated by suction, washed with dry acetonitrile and dried to give pure carbamoylides **3a-d** or carbamates **4a**-**d**. 

According to the above procedure the following compounds were obtained:

*4-Dimethylaminopyridinium 4-nitrophenylsulfonyl carbamoylide *(**3a**). Yield 56%; m.p. 175–177 °C (dec); IR (KBr) ν = 3099, 2937, 2683, 1691, 1645, 1578, 1520, 1349, 1294, 1257, 1215, 1159, 1072, 1078 cm^-1^; ^1^H-NMR (DMSO-*d_6_*) δ = 3.33 (s, 6H, CH_3_), 6.97 (d, 2H, CH, *J =* 7.2 Hz), 8.31 (d, 2H, CH, *J =* 8.7 Hz), 8.71 (d, 2H, CH, *J =* 8.7 Hz), 8.95 (d, 2H, CH, *J =* 7.2 Hz); Anal. Calcd. for C_14_H_14_ N_4_O_5_S (350.35) C, 47.99%; H, 4.03%; N, 15.99%; Found C, 47.72%; H, 3.87%; N, 15.83%.

*4-Dimethylaminopyridinium 2-chlorophenylsulfonyl carbamoylide* (**3b**). Yield: 54%; m.p. 112–115 °C; IR (KBr) ν = 3072, 2924, 1697, 1644, 1560, 1293, 1255, 1215, 1184, 1150, 1128, 1110, 1028 cm^-1^; ^1^H-NMR (DMSO-*d_6_*) δ = 3.16 (s, 6H, CH_3_), 6.96 (d, 2H, CH, *J =* 7.6 Hz), 7.60 (d, 1H, CH), 7.20-7.47 (m, 2H, CH), 7.95 (d, 1H, CH), 8.65 (d, 2H, CH, *J =* 7.6 Hz); Anal. Calcd. for C_14_H_14_ClN_3_O_3_S (339.80) C, 49.49%; H, 4.15%; N, 12.37%; Found C, 49.18%; H, 4.29%; N, 12.31%.

*4-Dimethylaminopyridinium naphth-2-ylsulfonyl carbamoylide* (**3c**). Yield: 53%; m.p. 153–156 °C (dec); IR (KBr) ν = 3087, 1707, 1648, 1572, 1289, 1253, 1217, 1150, 1090, 1065, 862, 834, 768 cm^-1^; ^1^H-NMR (DMSO*-d_6_*) δ = 3.22 (s, 6H, CH_3_), 6.95 (d, 2H, CH, *Ј =* 7.3 Hz), 7.60-7.69 (m, 2H, CH), 7.88-8.14 (m, 4H, CH), 8.43 (s, 1H, CH), 8.74 (d, 2H, CH, *Ј* = 7.3 Hz); Anal. Calcd. for C_18_H_17_N_3_O_3_S (355.41) C, 60.83%; H, 4.82%; N, 11.82%; Found C, 60.58%; H, 4.77%; N, 11.59%. 

*4-Dimethylaminopyridinium (4-chloropyridin-3-ylsulfonyl carbamoylide* (**3d**). Yield: 39%; m.p. 151–153 °C (dec); IR (KBr) ν = 3097, 2934, 1702, 1645, 1572, 1559, 1449, 1395, 1296, 1260, 1089, 842, 824, 766, 596 cm^-1^; ^1^H-NMR (DMSO-*d_6_*) δ = 3.25 (s, 6H, CH_3_), 7.00 (d, 2H, CH, *J* = 8.1 Hz), 7.64 (d, 1H, CH, *J* = 5.2 Hz), 8.64 (d, 1H, CH, *J* = 5.2 Hz), 8.73 (d, 2H, CH, *J* = 8.1 Hz), 9.07 (s, 1H, CH); Anal. Calcd. for C_13_H_13_ClN_4_O_3_S (340.79) C, 45.82%; H, 3.85%; N, 16.44%; Found C, 45.61%; H, 3.90%; N, 16.16%.

*4-(Dimethylamino)pyridinium (5-chlorothiophen-2-ylsulfonyl)(phenoxycarbonyl)amide* (**4a**). Yield: 54%; m.p. 144–146 °C (dec); IR (KBr) ν = 3077, 2924, 2611, 1964, 1645, 1562, 1409, 1295, 1262, 1210, 1186, 1173, 1024, 988, 924, 887, 807, 626 cm^-1^; ^1^H-NMR (DMSO-*d_6_*) δ = 3.18 (s, 6H, CH_3_), 6.96-7.38 (m, 8H, CH), 7.42 (d, 1H, CH, *J* = 3.9 Hz), 8.21 (d, 2H, CH, *J* = 7.6 Hz), 12.6 (bs, 1H, NH); Anal. Calcd. for C_18_H_18_ClN_3_O_4_S_2_ (439.94) C, 49.14%; H, 4.12%; N, 9.55%; Found C, 48.93%; H, 4.26%; N, 9.19%. 

*4-(Dimethylamino)pyridinium (benzothiazol-2-ylsulfonyl)(phenoxycarbonyl)amide* (**4b**). Yield: 73%; m.p. 135–138 °C (dec); IR (KBr) ν = 3060, 2924, 1685, 1646, 1559, 1271 cm^-1^; ^1^H-NMR (DMSO-*d_6_*) δ = 3.14 (s, 6H, CH_3_), 6.92-6.96 (m, 4H, CH), 7.02-7.12 (t, 1H, *J* = 7.4 Hz), 7.25-732 (t, 2H, CH, *J* = 7.4 Hz), 7.45-7.62 (m, 2H, CH), 8.04-8.16 (m, 2H, CH), 8.29 (d, 2H, CH, *J* = 7.6 Hz); Anal. Calcd. for C_21_H_20_N_4_O_4_S_2_ (456.54) C, 55.25%; H, 4.42%; N, 12.27%; Found C, 55.21%; H, 4.62%; N, 11.92%. Upon treatment of the above pyridinium salt with aqueous 1% HCl at room temperature the free carbamate **4b’** was obtained; m.p. 206-208 °C; IR (KBr) ν = 3007, 2851, 2799, 1758, 1458, 1222, 1096, 922, 767, 634 cm^-1^; ^1^H-NMR (DMSO-*d_6_*) δ = 6.75 (d, 2H, CH, *J* = 7.8 Hz), 7.16 (t, 1H, CH, *J* = 7.8 Hz), 7.35 (t, 2H, CH, *J* = 7.8 Hz), 7.60-7.72 (m, 2H, CH), 8.16-8.30 (m, 2H, CH), 8.35 (s, 1H, NH); Anal. Calcd. for C_14_H_10_N_2_O_4_S_2_ (334.37) C, 50.29%; H, 3.01%; N, 8.38%; Found C, 50.22%; H, 3.32%; N, 8.61%.

*4-(Dimethylamino)pyridinium (6-ethoxybenzothiazol-2-ylsulfonyl)(phenoxy-carbonyl)amide* (**4c**). Yield: 29.7%; m.p. 251–255 °C; IR (KBr) ν = 3223, 3066, 2924, 1681, 1645, 1557, 1296 cm^-1^; ^1^H-NMR (DMSO-*d_6_*) δ = 1.35 (t, 3H, CH_3_, *J* = 8.1 Hz), 3.14 (s, 6H, CH_3_), 4.09 (q, 2H, CH_2_, *J =* 8.1 Hz), 6.92-6.96 (m, 4H, CH), 7.05-7.15 (dd, 1H, CH, *J_ortho_* = 6.75, *J_meta_* = 2.3 Hz), 7.22-7.30 (t, 2H, CH, *J* = 7.4 Hz), 7.65 (d, 1H, CH, *J_meta_ =* 2.3 Hz), 7.93 (d, 1H, CH, *J_ortho_ =* 6.75 Hz), 8.19 (d, 2H, CH, *J* = 7.7 Hz); Anal. Calcd. for C_23_H_24_N_4_O_5_S_2_ (500.59) C, 55.18%; H, 4.83%; N, 11.19%; Found C, 54.97%; H, 5.11%; N, 10.98%. Upon treatment of the above pyridinium salt with aqueous 1% HCl the free carbamate **4c’** was obtained; m.p. 206-208 °C; IR (KBr) ν = 2989, 2859, 2787, 2663, 1756, 1599, 1485, 1375, 1262, 1217, 1168, 1080, 1042, 842, 759, 688, 627 cm^-1^; ^1^H-NMR (DMSO-*d_6_*) δ = 1.37 (t, 3H, CH_3_, *J* = 8.2 Hz), 4.12 (q, 2H, CH_2_, *J =* 8.2 Hz), 6.75 (m, 1H, CH), 7.00-7.45 (m, 5H, CH), 7.80-7.95 (m, 1H, CH), 8.00-8.15 (m, 1H, CH), 8.75 (bs, 1H, NH); Anal. Calcd. for C_16_H_14_N_2_O_5_S_2_ (378.42) C, 50.78%; H, 3.73%; N, 7.40%; Found C, 50.53%; H, 3.89%; N, 7.06%.

*4-(Dimethylamino)pyridinium (4-chloropyridin-3-ylsulfonyl)(phenoxycarbonyl)amide* (**4d**). Yield: 48%; m.p. 191–193 °C; IR (KBr) ν = 3215, 3082, 2922, 1688, 1646, 1561, 1397, 1263, 1191, 1149, 1028, 916, 887, 806, 797 cm^-1^; ^1^H-NMR (DMSO-*d_6_*) δ = 3.16 (s, 6H, CH_3_), 6.88-6.93 (m, 2H, CH), 6.96 (d, 2H, CH, *J* = 7.7 Hz), 7.06 (t, 1H, CH, *J* = 7.1 Hz), 7.22-7.30 (m, 2H, CH), 7.56 (d, 1H, CH, *J* = 5.3 Hz), 8.20 (d, 2H, CH, *J* = 7.7 Hz), 8.53 (d, 1H, CH, *J* = 5.3 Hz), 8.96 (s, 1H, CH), 12.60 (bs, 1H, NH); Anal. Calcd. for C_19_H_19_ClN_4_O_4_S (434.90) C, 52.47%; H, 4.40%; N, 12.88%; Found C, 52.19%; H, 4.71%; N, 12.60%. 

### 3.3. General procedure for preparation of arylsulfonyl ureas ***5*** and heteroarylsulfonyl ureas ***6***. Reaction of carbamoylides ***3a-c*** and carbamates ***4a-c*** with aliphatic and aromatic amines

A mixture of carbamoylide **3** or carbamate **4 **(2.8 mmol) and appropriate aliphatic or aromatic amine (3 mmol) in acetonitrile (10 mL) was heated at reflux for 10 min (in case of aliphatic) or 1 h (in case of aromatic) amine. The reaction mixture was cooled to room temperature and the solvent was evaporated under reduced pressure to dryness. The oily residue was suspended in methanol and treated with 10% aqueous hydrochloric acid. The sulphonylureas **5** or **6** that precipitated were separated by suction, washed with methanol and water and re-crystallized from suitable solvent.

According to the above procedure the following sulphonylureas were obtained: 

*N-(4-Nitrophenylsulfonyl)pyrrolidine-1-carboxamide* (**5a**). Yield: 17%; m.p. 187–193 °C (methanol); IR (KBr) ν = 3466, 3271, 3109, 2990, 2788, 1670, 1529, 1348, 1313, 1167, 1091, 1067 cm^-1^; ^1^H-NMR (DMSO-*d_6_*) δ = 1.60-1.90 (m, 4H, CH_2_CH_2_), 3.15-3.45 (m, 4H, N-CH_2_), 8.15 (d, 2H, CH, *J =* 5.2 Hz), 8.42 (d, 2H, CH, *J =* 5.2 Hz), 11.05 (bs, 1H, NH); Anal. Calcd. for C_11_H_13_N_3_O_5_S (299.30) C, 44.14%; H, 4.38%; N, 14.04%; Found C, 44.08%; H, 4.52; N, 13.96%. 

*N-4-Nitrophenylsulfonyl)-4-phenylopiperazine-1-carboxamide* (**5b**). Yield: 31%; m.p. 105–107 °C (ethanol); IR (KBr) ν = 3419, 3105, 2882, 1669, 1600, 1529, 1494, 1350, 1235, 1166, 1091 cm^-1^; ^1^H-NMR (DMSO-*d_6_*) δ = 2.90-3.20 (m, 4H, CH_2_), 3.30-3.60 (m, 4H, CH_2_), 6.80 (t, 1H, CH, *J* = 7.4 Hz), 6.90 (d, 2H, CH, *J* = 7.4 Hz), 7.20 (t, 2H, CH, *J* = 7.4 Hz), 8.50 (d, 2H, CH, *J =* 9.7 Hz), 8.43 (d, 2H, CH, *J =* 9.7 Hz), 11.45 (bs, 1H, NH); Anal. Calcd. for C_17_H_18_N_4_O_5_S (390.41) C, 52.30%; H, 4.65%; N, 14.35%; Found C, 52.05%; H, 4.83%; N, 14.16%. 

*N-4-Chlorophenylcarbamoyl)-4-nitrophenylsulfonamide* (**5c**). Yield: 42%; m.p. 165–170 °C (ethanol); IR (KBr) ν = 3438, 3351, 3112, 2890, 1715, 1607, 1542, 1351, 1158, 1086, 1039, 1013 cm^-1^; ^1^H-NMR (DMSO-*d_6_*) δ = 7.32 (d, 2H, CH, *J =* 8.8 Hz), 7.38 (d, 2H, CH, *J =* 8.8 Hz), 8.22 (d, 2H, CH, *J =* 8.8 Hz), 8.46 (d, 2H, CH, *J =* 8.8 Hz), 9.80 (bs, 1H, NH), 11.20 (bs, 1H, NH); Anal. Calcd. for C_13_H_10_ClN_3_O_5_S (355.75) C, 43.89%; H, 2.83%; N, 11.81%; Found C, 44.17%; H, 3.12%; N, 11.57%. 

*N-(4-Methoksyphenylcarbamoyl)-4-nitrophenylsulfonamide* (**5d**). Yield: 46%; m.p. 148–152 °C (ethanol); IR (KBr) ν = 3482, 3316, 3117, 3071, 3013, 2959, 2836, 1698, 1530, 1514, 1436, 1348, 1311, 1252, 1163, 1089, 1023 cm^-1^; ^1^H-NMR (DMSO-*d_6_*) δ = 3.68 (s, 3H, OCH_3_), 6.84 (d, 2H, CH, *J =* 9 Hz), 7.24 (d, 2H, CH, *J =* 9 Hz), 8.20 (d, 2H, CH, *J =* 9 Hz), 8.45 (d, 2H, CH, *J =* 9 Hz); 8.93 (bs, 1H, NH), 11.2 (bs, 1H, NH); Anal. Calcd. for C_14_H_13_N_3_O_6_S (351.33) C, 47.86%; H, 3.73%; N, 11.96%; Found C, 47.74%; H, 3.95%; N, 11.71%. 

*N-(2-Chlorophenylsulfonyl)pyrrolidine-1-carboxamide* (**5e**). Yield: 37%; m.p. 175–190 °C (methanol); IR (KBr) ν = 3400, 3271, 3102, 3068, 1694, 1575, 1487, 1436, 1255, 1236, 1185, 1168, 1132, 1115 cm^-1^; ^1^H-NMR (DMSO-*d_6_*) δ = 1.64-1-94 (m, 4H, CH_2_), 3.15-45 (m, 4H, N-CH_2_), 7.54-7.58 (m, 1H, CH), 7.60-7.68 (m, 2H, CH), 8.03 (d, 1H, CH, *J =* 7.7 Hz), 11.06 (s, 1H, NH); Anal. Calcd. for C_11_H_13_ClN_2_O_3_S (288.75) C, 45.75%; H, 4.54%; N, 9.70%; Found C, 45.61%; H, 4.82%; N, 9.47%. 

*2-Chloro-N-(pyrimidin-2-ylcarbamoyl)benzenesulfonamide* (**5f**). Yield: 68%; m.p. 178–182 °C; IR (KBr) ν = 3148, 3067, 2970, 2920, 1715, 1582, 1470, 1449, 1402, 1353, 1282, 1155, 1130, 1075, 1006, 914, 866, 812 cm^-1^; ^1^H-NMR (DMSO-*d_6_*) δ = 7.42-7.50 (m, 1H, CH), 7.65-7.80 (m, 3H, CH), 7.97-8.03 (d, 1H, CH, *J* = 7.6 Hz), 8.70-8.77 (m, 2H, CH), 10.69 (s, 1H, NH), 12.63 (bs, 1H, NH); Anal. Calcd. for C_11_H_9 _ClN_4_O_3_S (312.73); C, 42.25%; H, 2.90%; N, 17.92%; Found C, 41.89%; H, 3.27%; N, 17.65%. 

*N-(Naphthalene-2-ylsulfonyl)pyrrolidine-1-carboxamide*(**5g**). Yield: 48%; m.p. 203–208 °C (ethanol); IR (KBr) ν = 3266, 2975, 2870, 1692, 1441, 1328, 1157, 1057, 861, 748, 658 cm^-1^; ^1^H-NMR (DMSO-*d_6_*) δ = 1.60-1.90 (m, 4H, CH_2_), 3.15-3.45 (m, 4H, N-CH_2_), 7.62-7.75 (m, 2H, CH), 7.91-8.21 (m, 4H, CH), 8.57 (s, 1H, CH), 10.77 (s, 1H, NH); Anal. Calcd. for C_15_H_16_N_2_O_3_S (304.36) C, 59.19%; H, 5.30%; N, 9.20%; Found C, 58.98%; H, 5.43%; N, 9.01%. 

*N-(Naphthalene-2-ylsulfonyl)-4-phenylpiperazine-1-carboxamide* (**5h**). Yield: 98%; m.p. 119–123 °C (ethanol); IR (KBr) ν = 3277, 3059, 3016, 2959, 2936, 1676, 1484, 1338, 1252, 1162, 1125, 1070, 1021, 876, 745 cm^-1^; ^1^H-NMR (DMSO-*d_6_*) δ = 3.28-3.36 (m, 4H, CH_2_), 3.66-3.74 (m, 4H, CH_2_), 7.11-7.17 (m, 1H, CH), 7.30-7.40 (m, 4H, CH), 7.64-7.76 (m, 2H, CH), 7.92-8.22 (m, 4H, CH), 8.59 (s, 1H, CH), 11.35 (bs, 1H, NH); Anal. Calcd. for C_21_H_21_N_3_O_3_S (395.47) C, 63.78%; H, 5.35%; N, 10.63%; Found C, 63.56%; H, 5.50%; N, 10.33%.

*Ethyl 4-(naphthalen-2-ylsulfonylcarbamoyl)piperazine-1-carboxylate* (**5i**). Yield: 48%; m.p. 143–147 °C; IR (KBr) ν = 3196, 3060, 2978, 2930, 2868, 1709, 1677, 1469, 1441, 1257, 1230, 1163, 1072 cm^-1^; ^1^H-NMR (DMSO-*d_6_*) δ = 1.16 (t, 3H, CH_3_, *Ј* = 7 Hz), 3.20-3.42 (m, 8H, CH_2_), 4.02 (q, 2H, CH_2_, *Ј* = 7 Hz), 7.67-7.73 (m, 2H, CH), 7.88-7.93 (m, 1H, CH), 8.02-8.21 (m, 3H, CH), 8.55 (s, 1H, CH), 11.20 (bs, 1H, NH); Anal. Calcd. for C_18_H_21_N_3_O_5_S (391.44) C, 55.23%; H, 5.41%; N, 10.73%; Found C, 54.94%; H, 5.72%; N, 10.66%. 

*N-(Pyrimidin-2-ylcarbamoyl)naphthalene-2-sulfonamide* (**5j**). Yield: 68%; m.p. 177–182 °C; IR (KBr) ν = 3148, 3067, 2971, 2920, 1716, 1583, 1471, 1449, 1354, 1283, 1156, 1075 cm^-1^; ^1^H-NMR (DMSO-*d_6_*) δ = 7.20-7.24 (m, 1H, CH), 7.66-7.78 (m, 2H, CH), 7.98-8.26 (m, 4H, CH), 8.70-8.72 (m, 3H, CH), 10.70 (s, 1H, NH), 12.65 (bs, 1H, NH); Anal. Calcd. for C_15_H_12_N_4_O_3_S (328.35) C, 54.87%; H, 3.68%; N, 17.06%; Found C, 55.02%; H, 3.84%; N, 16.81%. 

*N-(5-Chlorothiophen-2-ylsulfonyl)indoline-1-carboxamide* (**6a**). Yield: 73%; m.p. 182–184 °C; IR (KBr) ν = 3635, 3450, 2920, 1659, 1486, 1464, 1400, 1348, 1165, 1137, 1091, 998, 808, 871, 679, 620, 604 cm^-1^; ^1^H-NMR (DMSO-*d_6_*) δ = 3.11 (t, 2H, CH_2_, *J* = 8.4 Hz), 4.03 (t, 2H, CH_2_, *J* = 8.4 Hz), 6.92-7.01 (m, 1H, CH), 7.08-7.23 (m, 2H, CH), 7.27 (d, 1H, CH, *J* = 4.2 Hz), 7.69 (d, 1H, CH, *J* = 4.2 Hz), 10.9 (bs, 1H, NH); Anal. Calcd. for C_13_H_11_ClN_2_O_3_S_2_ (342.82) C, 45.55%; H, 3.23%; N, 8.17%; Found C, 45.38%; H, 3.59%; N, 7.93%. 

*N-(5-Chlorothiophen-2-ylsulfonyl)-3,4-dihydroisoquinoline-2(1H)-carboxamide* (**6b**). Yield: 61%; m.p. 169–172 °C; IR (KBr) ν = 3107, 3020, 2896, 2805, 2751, 1646, 1481, 1410, 1344, 1235, 1167, 1001, 757, 616, 572 cm^-1^; ^1^H-NMR (DMSO-*d_6_*) δ = 2.81-2.82 (m, 2H, CH_2_), 3.59-3.61 (m, 2H, CH_2_), 4.53 (s, 2H, CH_2_), 7.16-7.20 (m, 4H, CH), 7.25 (d, 1H, CH, *J* = 3.9 Hz), 7.64 (d, 1H, CH, *J* = 3.9 Hz), 11.44 (bs, 1H, NH); Anal. Calcd. for C_14_H_13_ClN_2_O_3_S_2_ (356.85) C, 47.12%; H, 3.67%; N, 7.85%; Found C, 46.84%; H, 3.73%; N, 7.57%.

*5-Chloro-N-(2,3-dihydro-1H-inden-1-ylcarbamoyl)thiophene-2-sulfonamide* (**6c**). Yield: 49%, m.p. 144–148 °C; IR (KBr) ν = 3353, 3094, 1687, 1650, 1541, 1466, 1409, 1367, 1168, 995, 752, 679 cm^-1^; ^1^H-NMR (DMSO-*d_6_*) δ = 1.71-1.86 (m, 1H, CH), 2.30-2.48 (m, 1H, CH), 2.68-2.96 (m, 2H, CH), 5.00-5.18 (m, 1H, CH, *J* = 8.0 Hz), 6.99 (d, 1H, NH, *J* =8.0 Hz), 7.10-7.24 (m, 4H, CH), 7.29 (d, 1H, CH, *J* = 4.1 Hz), 7.65 (d, 1H, CH, *J* = 4.1 Hz), 10.90 (bs, 1H, NH); Anal. Calcd. for C_14_H_13_ClN_2_O_3_S_2_ (356.85) C, 47.12%; H, 3.67%; N, 7.85%; Found C, 46.97%; H, 3.95%; N, 7.82%. 

*N-(Benzothiazol-2-ylsulfonyl)pyrrolidine-1-carboxamide* (**6d**). Yield: 80%; m.p. 220–222 °C; IR (KBr) ν = 3277, 2877, 1690, 1454, 1168, 1056, 860, 765, 625 cm^-1^; ^1^H-NMR (DMSO-*d_6_*) δ = 1.60-1.95 (m, 4H, CH_2_), 3.25-3.65 (m, 4H, CH_2_), 7.61-7.73 (m, 2H, CH), 8.16-8.32 (m, 2H, CH), 11.80 (bs, 1H, NH); Anal. Calcd. for C_12_H_13_N_3_O_3_S_2_ (311.38); C, 46.29%; H, 4.21%; N, 13.49%; Found C, 45.97%; H, 4.53%; N, 13.42%. 

*N-(Benzothiazol-2-ylsulfonyl)thiazolidine-1-carboxamide* (**6e**). Yield: 79%; m.p. 240–241 °C; IR (KBr) ν = 3068, 2887, 2791, 1681, 1455, 1181, 1385, 1092, 859, 626 cm^-1^; ^1^H-NMR (DMSO-*d_6_*) δ = 2.98-3.04 (m, 2H, CH_2_), 3.59-3.63 (m, 2H, CH_2_), 4.42 (s, 2H, CH_2_), 7.61-7.74 (m, 2H, CH), 8.16-8.22 (m, 1H, CH), 8.26-8.33 (m, 1H, CH), 11.20 (bs, 1H, NH); Anal. Calcd. for C_11_H_11_N_3_O_3_S_3_ (329.42) C, 40.11%; H, 3.37%; N, 12.76%; Found C, 39.87%; H, 3.42%; N, 12.40%.

*N-(4-Chlorophenylcarbamoyl)benzothiazole-2-sulfonamide* (**6f**). Yield: 81%, m.p. 182–184 °C; IR (KBr) ν = 3281, 1709, 1597, 1532, 1367, 1166, 1028, 923, 673 cm^-1^; ^1^H-NMR (DMSO-*d_6_*) δ = 7.28 (d, 1H, CH, *J* = 7.6 Hz,), 7.38 (d, 2H, CH, *J* = 7.6 Hz), 7.62-7.72 (m, 2H, CH), 8.16-8.23 (m, 1H, CH), 8.25-8.34 (m, 1H, CH), 9.39 (bs, 1H, NH), 12.6 (bs, 1H, NH); Anal. Calcd. for C_14_H_10_ClN_3_O_3_S_2_ (367.83) C, 45.71%; H, 2.74%; N, 11.42%; Found C, 45.34%; H, 3.11%; N, 11.10%. 

*N-(Pyrimidin-2-ylcarbamoyl)benzothiazole-2-sulfonamide* (**6g**). Yield: 70%; m.p. 312–314 °C; IR (KBr) ν = 3067, 2972, 2919, 1646, 1583, 1449, 1374, 1161, 1028, 917, 761 cm^-1^; ^1^H-NMR (DMSO-*d_6_*) δ = 7.27-7.32 (m, 1H, CH), 7.56-7.69 (m, 2H, CH), 8.10-8.16 (m, 1H, CH), 8.19-8.27 (m, 1H, CH), 8.73-8.76 (m, 2H, CH), 11.43 (bs, 2H, NH); Anal. Calcd. for C_12_H_9_N_5_O_3_S_2_ (335.36) C, 42.98%; H, 2.70%; N, 20.88%; Found C, 42.71%; H, 3.02%; N, 20.57%. 

*N-(6-Ethoxybenzothiazol-2-ylsulfonyl)-3,4-dihydroisoquinoline-2(1H)-carboxamide* (**6h**). Yield: 87%; m.p. 203–206 °C; IR (KBr) ν = 3145, 3088, 2956, 2876, 1648, 1555, 1432, 1386, 1145, 1128, 1034, cm^-1^; ^1^H-NMR (DMSO-*d_6_*) δ = 1.37 (t, 3H, CH_3_, *J* = 7.8 Hz), 2.80 (t, 2H, CH_2_, *J* = 6.8 Hz), 3.60 (t, 2H, CH_2_, *J* = 6.8 Hz), 4.12 (q, 2H, CH_2_, *J* = 7.8 Hz), 4.51 (s, 2H, CH_2_), 7.05-7.2 (m, 4H, CH), 7.23 (dd, 1H, CH, *J_ortho_* = 6.8 Hz, *J*_meta_ = 2.0 Hz), 7.80 (d, 1H, CH, *J_ortho_* = 6.8 Hz), 8.05 (d, 1H, CH, *J_meta_* = 2.0 Hz), 11.30 (bs, 1H, NH); Anal. Calcd. for C_19_H_19_N_3_O_4_S_2_ (417.50) C, 54.66%; H, 4.59%; N, 10.06%; Found 54.56%; H, 4.85%; N, 10.03%. 

*N-(4-Chlorophenylcarbamoyl)-6-ethoxybenzothiazol-2-ylsulfonamide* (**6i**). Yield: 75%; m.p. 238–240 °C; IR (KBr) ν = 3326, 3194, 3088, 2967, 2902, 2887, 1642, 1558, 1442, 1390, 1148, 1148, 1142, 1044 cm^-1^; ^1^H-NMR (DMSO-*d_6_*) δ = 1.37 (t, 3H, CH_3_, *J* = 7.7 Hz), 4.16 (q, 2H, CH_2_, *J* = 7.7 Hz), 7.26 (dd, 1H, CH, *J_ortho_* = 6.9 Hz, *J*_meta_ = 2.1 Hz), 7.30 (d, 2H, CH, *J* = 7.0 Hz), 7.38 (d, 2H, CH, *J* = 7.0 Hz), 7.80 (d, 1H, CH, *J* = 2.1 Hz), 8.06 (d, 1H, CH, *J* = 6.9 Hz), 9.30 (s, 1H, NH), 11.80 (bs, 1H, NH); Anal. Calcd. for C_16_H_14_ClN_3_O_4_S_2_ (411.88) C, 46.66%; H, 3.43%; N, 10.20%; Found C, 46.73%; H, 3.68%; N, 9.83%. 

### 3.4. Preparation of 3-(indolin-1-yl)-pyrido[3,4-e][1,4,3]oxathiazine 1,1-dioxide ***8***

A mixture of carbamoylide **3d** or pyridinium carbamate **4d** (2.8 mmol) and indoline (3 mmol) in acetonitrile (10 mL) was heated at reflux for 3 h under reflux. The reaction mixture was cooled to room temperature and solvent was evaporated under reduced pressure to dryness. The crude residue triturated with methanol and water to give product **8** which was then separated by suction, washed with methanol and water and purified by crystallization from DMF. Yield: 33% and 29%, respectively; m.p. 248–250 °C; IR (KBr) ν = 2919, 1633, 1586, 1488, 1472, 1315, 1301, 1173, 1151, 1104, 1011, 765, 595 cm^-1^; ^1^H-NMR (DMSO-*d_6_*) δ = 3.25 (t, 2H, CH_2_, *J* = 7.8 Hz), 4.39 (t, 2H, CH_2_, *J* = 7.8 Hz), 7.18-7.21 (m, 1H, CH), 7.34-7.39 (m, 2H, CH), 7.62 (d, 1H, CH, *J* = 5.4 Hz), 8.07-8.09 (m, 1H, CH), 8.88 (d, 1H, CH, *J* = 5.4 Hz), 9.13 (s, 1H, CH); Anal. Calcd. for C_14_H_11_N_3_O_3_S (301.32) C, 55.80%; H, 3.68%; N, 13.95%; Found C, 55.74%; H, 3.78%; N, 14.13%.

### 3.5. X-ray structure analysis

The diffraction data were collected with a KumaCCD diffractometer using graphite monochromated Mo *K_α _*radiation. The intensity data were collected and processed using Oxford Diffraction CrysAlis Software [17]. The crystal structures were solved by direct methods with the program SHELXS-97 [18] and refined by full-matrix least-squares method on F^2^ with SHELXL-97 [18]. 

*Crystal data for C_14_H_9_N_2_O_4_S_2_·C_7_H_11_N_2_* (**4b**): Triclinic, space group *P-1, a* = 11.2168(5), *b* = 12.0651(6), *c* = 16.3837(8) Å, α = 79.042(4), *β =* 85.852(4), γ = 83.798(4)°, *V* = 2161.02(18) Å^3^, *Z* = 4, *d_x_* = 1.403 g.cm^-3^, *T* =130K. Data were collected for a crystal with dimensions 0.4x0.4x0.3 mm. Final R indices for 5697 reflections with I>2σ(I) and 582 refined parameters are: R_1 _= 0.0319, wR_2 _= 0.0764 (R_1_ = 0.0490, wR_2_ = 0.0864 for all 7589 data). The benzothiazole ringof one of the symmetry independent molecules is disordered over two strongly overlapping positions.

*Crystal data for C_16_H_13_N_2_O_5_S_2_·C_7_H_11_N_2_* (**4c**): Monoclinic, space group *P*2_1_*/n, a* = 12.3446(2), *b* = 12.5528(2), *c* = 15.6694(3) Å, *β =* 99.7467(18)°, *V* = 2393.07(7) Å^3^, *Z* = 4, *d_x_* = 1.389 g.cm^-3^, *T* = 130K. Data were collected for a crystal with dimensions 0.5x0.5x0.1 mm. Final R indices for 4467 reflections with I > 2σ(I) and 314 refined parameters are: R_1_ = 0.0291, wR_2_ = 0.0721 (R_1_ = 0.0391, wR_2_ = 0.0832 for all 5320 data). 

Crystallographic data for compounds **4b **and**4c **have been deposited with Cambridge Crystallographic Data Centre (CCDC deposition numbers CCDC 742624-742625). Copies of the data can be obtained upon request from CCDC, 12 Union Road, Cambridge CB2 1EZ, UK, quoting the deposition numbers.

### 3.6. In vitro cytotoxicity assay

The following primary screening of the new compounds was done to indicate whether a substance possesses enough activity at the concentration of 20 μM to inhibit human tumor cell growth by 50% (GI_50_ < 20 μM). 

The *in vitro* cytotoxic activity of all arylsulfonylureas **5a**-**j** and heteroarylsulfonylureas **6a**-**i** were evaluated [19,20] using human urinary bladder cancer 5637, small cell lung cancer A-427 and large cell lung cancer LCLC-103H cell lines. The assay was carried out in 96-well microtiter plates. When the cells were putted into the plates after 24 h cells were treated with appropriate drug solutions. The cytotoxic effects of the compounds were measured after a 96 h continuous exposure to the substances. The cell growth inhibition values were estimated by staining the adherent cells with crystal violet. Only viable cells remained attached to the plastic surface of the wells and bind the dye. The unbound dye was washed out with water and stain remaining in the wells was redissolved with 70% ethanol. Finally, the optical density (OD) was measured with a microplate reader set at λ = 570 nm. 

## 4. Conclusions

The current work has addressed the use of environmentally non hazardous aryl(heteroaryl)sulfonamides and diphenyl carbonate (DPC) in the synthesis of 4-dimethylamino-pyridinium *N*-[aryl(heteroaryl)sulfonyl] carbamoylides of type **3**, the stable and easy-to-handle substitutes of aryl(heteroaryl)sulfonyl isocyanates. The comparison of the existing literature methods for the preparation of arylsulfonyl/heteroaryl carbamates and ureas from arylsulfonamides suggests that the DPC/DMAP approach is superior. Some of the advantages include mild reaction conditions, the ease of preparation and product separation and the extended shelf-life of the parent ylides. Moreover, a very high reactivity of pyridinium carbamoylides renders them suitable for the syntheses of arylsulfonyl ureas. It should be emphasized, however, that in the above procedure diphenyl carbonate (DPC) could not be replaced by the less reactive diethyl or dimethyl carbonates.

It has been also found that the course of the reaction between sulphonamide and DPC/DMAP couple depends on p*K*_a_ of the substrate. Thus, arylsulphonamides with p*K*_a_ in the range of 9-10 give the desired carbamoylides **3**, while the more acidic heteroarylsulfonamides (p*K*_a_ ~ 8) react preferentially with the formation of pyridinium salts of the intermediary formed carbamates **4**.

Although none of the compounds described in this work exhibited pronounced cytotoxic activity against selected human tumor cell lines, yet the biological potential of these derivatives incorporating aryl(heteroaryl)sulfonylurea pharmacophoric group presented in Introduction remains to be explored.
